# Comparison of traditional regression modeling vs. AI modeling for the prediction of dental caries: a secondary data analysis

**DOI:** 10.3389/froh.2024.1322733

**Published:** 2024-05-24

**Authors:** Priya Dey, Chukwuebuka Ogwo, Marisol Tellez

**Affiliations:** Maurice H Kornberg School of Dentistry, Oral Health Sciences, Temple University, Philadelphia, PA, United States

**Keywords:** machine learning, dental caries, prediction, Artificial Intelligence, traditional statistical

## Abstract

**Introduction:**

There are substantial gaps in our understanding of dental caries in primary and permanent dentition and various predictors using newer modeling methods such as Machine Learning (ML) algorithms and Artificial Intelligence (AI). The objective of this study is to compare the accuracy, precision, and differences between the caries predictive capability of AI vs. traditional multivariable regression techniques.

**Methods:**

The study was conducted using secondary data stored in the Temple University Kornberg School of Dentistry electronic health records system (axiUm) of pediatric patients aged 6–16 years who were patients on record at the Pediatric Dentistry Clinic. The outcome variables considered in the study were the decayed–missing–filled teeth (DMFT) and the decayed–extracted–filled teeth (deft) scores. The predictors included age, sex, insurance, fluoride exposure, having a dental home, consumption of sugary meals, family caries experience, having special needs, visible plaque, medications reducing salivary flow, and overall assessment questions.

**Results:**

The average DMFT score was 0.85 ± 2.15, while the average deft scores were 0.81 ± 2.15. For childhood dental caries, XGBoost was the best performing ML algorithm with accuracy, sensitivity. and Kappa as 81%, 84%, and 61%, respectively, followed by Support Vector Machine and Lasso Regression algorithms, both with 84% specificity. The most important variables for prediction found were age and visible plaque.

**Conclusions:**

The machine learning model outperformed the traditional statistical model in the prediction of childhood dental caries. Data from a more diverse population will help improve the quality of caries prediction for permanent dentition where the traditional statistical method outperformed the machine learning model.

## 1 Introduction

Dental caries is the most prevalent oral disease affecting children and adolescents resulting in deterioration of oral health ultimately leading to tooth loss (1,2). According to the Centers for Disease Control and Prevention, about 23% of children have dental caries. Hence, it is becoming increasingly important to manage dental caries at an early age as early detection of the disease allows for a more preventive medical management (3).

Globally, the decayed–missing–filled teeth (DMFT) index has been widely accepted as a population-based measure of dental caries. DMFT along with other predictors such as demographics, potential risk, and protective factors have been strategized for individual risk assessment and as targets for caries management (3).

Utilization of various standardized checklists for the assessment of caries risk has been done for many decades now. These tools used for estimating caries risk are used in day-to-day practice for advising in the clinical decision-making process using individually tailored disease prevention (4). The foundation for successful caries management is conducting caries risk assessment (CRA). Some of the well-known and widely used Caries Risk Assessment systems include the Caries Management by Risk Assessment (CAMBRA) form, the Cariogram, the American Dental Association (ADA) checklist, and the American Academy of Pediatric Dentistry (AAPD) form (5).

Traditional multivariable regression techniques are age-old reliable methods used for caries prediction, but these traditional regression models face limitations based on the principles that need to be followed for regression modeling. These include satisfying assumptions such as independence, following normal distribution among other assumptions. Multicollinearity effect among variables cannot be as strongly studied when using regression models (6). Hence, there is a need to study the prediction of dental caries using stronger modeling techniques. Conversely, some of the newer adopted techniques include Artificial Intelligence (AI) based on Machine Learning (ML) modeling, which in comparison with the traditional approaches have shown to have higher prediction accuracy and are likely to contribute significantly to the diagnostic process. ML is a subset of AI that utilizes algorithms trained on datasets to produce models that can perform complex tasks by reducing over-/under-fitting of models (1). Furthermore, AI can analyze data with a variety of features that cannot be handled by traditional regression techniques such as the ability to test the performance of a model itself (4).

## 2 Materials and methods

No prior studies comparing AI vs. traditional modeling have been conducted for predicting caries outcomes and using risk factor information from a standardized CRA system. Therefore, the aim of this study was to compare the accuracy, precision, and differences between the caries predictive capability of AI vs. traditional multivariable regression techniques in a sample of pediatric patients attending the Temple University Maurice H Kornberg School of Dentistry (TUKSoD), located in Philadelphia, Pennsylvania, United States.

This secondary data analysis research study was approved by the Institutional Review Board (IRB) at Temple University. The secondary data collected belongs to pediatric patients attending the clinics at the Dental School.

### 2.1 Database and inclusion/exclusion criteria

Data collection involved requesting secondary data stored in the school's electronic health records system (axiUm) of patients aged 6–16 years who were patients on records at the Pediatric Dentistry Clinic. The data retrieved from axiUm were from 1 March 2021 to 1March 2022. Other participants younger than 6 or older than 17 years of age or emergency patients were excluded from the study.

### 2.2 Outcomes and predictors

The outcome variables considered in the study were DMFT and the decayed–extracted–filled teeth (deft) scores. Individuals with DMFT/deft = 0 were grouped as “having no caries” and those with DMFT/deft score >0 were grouped as “having caries.” The predictors included demographic variables such as age, sex (male, female, and transgender), and insurance (cash/no insurance, private insurance, Medicaid, Ryan White, and Medicare). The potential protective factors included fluoride exposure (yes/no) and having a dental home (yes/no), while potential risk factors included consumption of sugary meals (primarily at meals and frequently or for prolonged periods between meals), family caries experience (none in 24 months, lesions in last 7–23 months, and lesions in last 6 months), having special needs [no, yes (over age 14), and yes (age 6–14)], medications reducing salivary flow (yes/no), and overall assessment questions gauging if participants were given any additional education (yes/no) or received fluoride application (yes/no) (Appendix A).

### 2.3 Data analysis

Descriptive statistics such as mean ± standard deviation (SD), range, and frequency were generated for all the continuous variables. DMFT and deft scores were stratified by the predictors. Bivariate tests were conducted and included Pearson correlation coefficients between DMFT/deft scores and selected continuous variables, while independent *T*-tests were done for predictors with one category. Analysis of variance (ANOVA) was performed on those predictors with more than two categories. DMFT and deft were dichotomized into categorical variables and appropriate Chi-square tests were conducted between various categorical predictor variables. The significance level was set at *p* < 0.05.

A logistic regression model was used to test for associations between predictor variables while adjusting for confounders on the categorical outcome variable. The traditional negative binomial regression model and logistic model were compared with AI modeling (Logistic Regression, XGBoost, Support Vector Machine, and Lasso Regression). The datasets were divided using resampling techniques based on fivefold cross-validation. In using this technique, 80% of the data were used for training and 20% were used for testing at each stage of the resampling. The machine learning space allowed for models to be trained, which allows them to learn about each type of unit. After the training phase, the model was assessed on the test data to predict the kind of unlabeled data. To analyze the findings, supervised learning models such as Logistic Regression, XGBoost, Support Vector Machine, and Lasso Regression were applied. Hyperparameter tuning was performed by adjusting the nrounds = 10, eta = 0.2, and objective = “binary:logistic.” The predictive accuracy, precision, area under the receiver operating characteristic curve (AUC-ROC), specificity, and sensitivity of these AI models were also compared.

## 3 Results

### 3.1 Descriptive characteristics of data

The total number of subjects included in the study was 3,586. The average DMFT score was 0.85 ± 2.15, while the average deft scores were 0.81 ± 2.15. The mean age of the selected participants was 12 years. The sample comprised mostly girls (53.9%) who had Medicaid insurance (82.29%). The potential protective factors included those exposed to fluoride (46.8%) and having a dental home (36.42%). The potential risk factors included frequent consumption of sugary meals (25.2%), family caries experience (9%), special healthcare needs (40%), medication reducing salivary flow (1.7%), visible plaque (33%), unusual tooth morphology (7%), exposed root surface (2.4%), orthodontic appliances (4.2%), and severe xerostomia (0.16%). About 10.6% of the patients declined additional fluoride application, while 39.5% of them received additional caries prevention educational material from their dentists (Table 1).

**Table 1:** Descriptive analysis (*n* = 3,586).

Variables		Mean ± SD	
DMFT score		0.85 ± 2.15	
deft score		0.81 ± 2.15	
Age		12.20 ± 3.10	
		*N*	%
DMFT	Score = 0	2,698	75
	Score > 0	888	25
deft	No	2,950	82.26
	Yes	636	17.74
Sex	Male	1,650	46
	Female	1,935	53.9
	Transgender	1	0.01
Insurance	Cash/No insurance	253	7.05
	Private	379	10.56
	Medicaid	2,951	82.29
	Ryan White	—	—
	Medicare	3	0.08
Protective factors			
Fluoride exposure	No	107	2.9
	Yes	1,680	46.8
	Unanswered	1,799	50.1
Dental home	No	458	12.7
	Yes	1,306	36.42
	Unanswered	1,822	50.8
Risk factors			
Sugary foods or drinks	Primarily at meals	873	24.3
	Frequent or prolonged intake	913	25.4
	Unanswered	1,800	50.2
Caries experience of family	None in 24 months	1,016	28.3
	Lesions in last 7–23 months	348	9.7
	Lesions in last 6 months	154	4.2
	Unanswered	2,068	57.6
Special healthcare needs	No	1,680	46.8
	Yes (over age 14)	25	40.69
	Yes (age 6–14)	68	1.8
	Unanswered	1,813	50.59
Medications that reduce salivary flow	No	1,710	47.6
	Yes	62	1.7
	Unanswered	1,814	50.5
Visible plaque	No	422	11.7
	Yes	1,214	33.8
	Unanswered	1,950	54.3
Unusual tooth morphology, that compromises oral hygiene	No	1,388	38.70
	Yes	256	7.13
	Unanswered	1,942	54.1
Exposed root surfaces present	No	1,621	45.2
	Yes	25	2.4
	Unanswered	1,940	54.4
Dental/orthodontic appliances (fixed or removable)	No	1,502	41.8
	Yes	149	4.1
	Unanswered	1,935	53.9
Severe dry mouth (Xerostomia)	No	1,641	45.76
	Yes	6	0.16
	Unanswered	1,939	54.0
Overall assessment			
Was caries prevention educational material provided to the patient?	No	158	4.4
	Yes	1,419	39.5
	Unanswered	2,009	56.0
Patient has been offered fluoride application and declines to receive it in the clinic	No	1,073	29.9
	Yes	380	10.6
	Unanswered	2,133	59.4

### 3.2 Bivariate analysis

DMFT was statistically significantly associated with age (*p* < 0.001), gender (mean ± SD, male DMFT: 0.75 ± 1.97, female DMFT: 0.95 ± 2.29, *p*-value <0.05], and sugary meal consumption (mean ± SD, primarily at meals DMFT: 1.43 ± 2.7, frequently or for prolonged periods between meals DMFT: 1.75 ± 2.76, *p* < 0.05) (Table 2).

**Table 2:** Bivariate analysis between DMFT and selected risk and protective factors (*n* = 912).

DMFT by	** **	Mean ± SD	*P*-value
	Age		**<0**.**01**
Sex	Male	0.75 ± 1.97	**0**.**01**
	Female	0.95 ± 2.29	
Insurance	Cash/No insurance	0.52 ± 1.62	0.07
	Private	0.60 ± 1.78	
	Medicaid	0.91 + 1.22	
Fluoride exposure	No	1.96 + 2.7	0.91
	Yes	1.57 + 2.7	
Dental Home	No	1.72 + 3.1	0.66
	Yes	1.55 + 2.62	
Sugary meals	Primary at meals	1.43 + 2.7	<**0**.**05**
	Frequent or prolonged intake between meals	1.75 + 2.76	
Family caries experience	None in 24 months	1.32 + 2.30	0.92
	Lesions in last 7–23 months	1.54 + 2.6	
	Lesions in last 6 months	1.58 + 2.77	
Special healthcare needs	No	1.60 + 2.76	0.18
	Yes (over age 14)	2.64 + 3.75	
	Yes (age 6–14)	1 + 1.77	
Medication reducing salivary flow	No	1.59 + 2.76	0.17
	Yes	1.85 + 2.51	
Plaque	No	1.19 + 2.31	**=0**.**05**
	Yes	1.81 + 2.96	
Unusual tooth morphology	No	1.50 + 2.58	0.27
	Yes	2.39 + 3.67	
Exposed root surface	No	1.62 + 2.78	0.29
	Yes	2.76 + 3.91	
Orthodontic appliance	No	1.62 + 2.77	0.29
	Yes	1.91 + 3.19	
Severe xerostomia	No	1.64 + 2.8	0.46
	Yes	5.00 + 4.19	
Educational provision given	No	1.74 + 3.33	0.97
	Yes	1.66 + 2.7	
Additional fluoride denial	No	1.68 + 2.77	0.48
	Yes	1.47 + 2.48	

Bold values are statistically significant.

deft was statistically significantly associated with age (*p* < 0.001), family caries experience (mean ± SD, lesions in 7–23 months deft: 1.85 + 2.88 and lesions in past 6 months deft: 2.17 ± 3.20, *p* < 0.05), having unusual tooth morphology (mean ± SD, none deft: 1.47 ± 2.67 and having unusual tooth morphology deft score: 0.98 ± 2.44, *p* < 0.05), and use of orthodontic appliance (mean ± SD, no: 1.43 + 2.64 and using appliance deft score: 0.95 + 2.57, *p* < 0.05) (Table 3).

**Table 3:** deft stratified on the predictors (*n* = 912).

deft by	** **	Mean ± SD	*P*-value
	Age		**<0**.**001**
Sex	Male	0.93 + 2.7	0.15
	Female	0.70 + 2.2	
Insurance	Cash/No insurance	0.46 + 1.72	0.41
	Private	0.58 + 1.77	
	Medicaid	0.87 + 2.22	
Fluoride exposure	No	1.19 + 2.42	0.13
	Yes	1.46 + 2.70	
Dental Home	No	1.34 + 2.73	0.62
	Yes	1.48 + 2.67	
Sugary meals	Primarily at meals	1.13 + 2.29	0.059*
	Frequent or prolonged intake between meals	1.75 + 2.99	
Family caries experience	None in 24 months	1.33 + 2.55	**<0**.**001**
	Lesions in 7–23 months	1.85 + 2.88	
	Lesions in last 6 months	2.17 + 3.20	
Special healthcare needs	No	1.44 + 2.69	0.08
	Yes (over age 14)	0.28 + 1.20	
	Yes (age 6–14)	1.86 + 2.83	
Medication reducing salivary flow ghcQ4	No	1.46 + 2.70	0.35
	Yes	0.93 + 2.08	
Plaque	No	1.23 + 2.43	0.10
	Yes	1.47 + 2.72	
Unusual tooth morphology	No	1.47 + 2.67	**=0**.**04**
	Yes	0.98 + 2.44	
Exposed root surface	No	1.38 + 2.62	0.89
	Yes	1.32 + 2.35	
Orthodontic appliance	No	1.43 + 2.64	**<0**.**001**
	Yes	0.95 + 2.57	
Educational provision given	No	1.24 + 2.45	0.31
	Yes	1.38 + 2.61	
Additional fluoride denial	No	1.40 + 2.63	0.24
	Yes	1.35 + 2.51	

*p* = 0.05 cannot be rejected or accepted.

Bold values are statistically significant.

Dichotomizing DMFT and deft into categorical variables showed that DMFT was significantly associated with predictors such as age, consumption of sugary meals, and visible plaque. While deft was significantly associated with predictor variables such as age, family caries experience, special healthcare needs, and dental appliance use.

### 3.3 Multivariable analysis

#### 3.3.1 Traditional logistic regression

As presented in Table 4, after careful consideration of the multicollinearity effect, interaction terms, and consideration of all fitted models, we found that holding all other variables in the model constant, age was found significantly associated with DMFT [odds ratio (OR) = 0.89, 95% CI (0.27–0.38)]. As age increased, the odds of having dental caries experience decreased by 11%. In addition, having insurance decreased the odds of having dental caries by 12% [OR = 0.88, 95% CI (0.032–0.62)], and those who frequently consumed sugary meals and drinks were 1.19 times more likely to have dental caries in comparison with those who did not consume sugary meals [OR = 1.19, 95% CI (0.15–0.74)]. Lastly, those who had visible plaque were 1.18 times more likely to have dental caries in comparison with those who did not have plaque [OR = 1.18, 95% CI (0.09–0.78)].

**Table 4:** Logistic regression (DMFT and deft).

Model	** **	OR (95% CI)	CI (95%) LL	CI (95%) UL
DMFT	Age	0.89	**0**.**27**	**0**.**38**
	Sex (M)	0.96	**−0**.**63**	**−0**.**05**
	Insurance (No)	0.88	**0**.**032**	**0**.**62**
	Fluoride exposure (Yes)	1.49	−0.15	1.26
	Sugary meals (No)	1.19	**0**.**15**	**0**.**74**
	Dental Home (No)	0.74	−0.06	0.61
	Plaque (No)	1.18	**0**.**09**	**0**.**78**
	Dental appliances causing xerostomia (No)	0.97	−0.89	0.18
	Patient offered fluoride and declines (Yes)	0.68	−0.58	0.07
deft	Age	1.65	**−0**.**69**	**−0**.**53**
	Sugary meals (Frequent consumption)	0.73	−0.05	0.65
	Special needs yes (age 6–14)	2.66	**0**.**02**	**0**.**20**
	Plaque (No)	1.31	**0**.**05**	**0**.**88**
	Patient offered fluoride and declines (No)	1.29	−0.75	0.04

Reference is mentioned in bracket and significant variables are in bold.

As presented in Table 4, only age [OR = 1.65, 95% CI (−0.69 to −0.53)], special needs [OR = 2.66, 95% CI (0.02–0.20)], and visible plaque [OR = 1.31, 95% CI (0.05–0.88)] were found to be significant predictors of dental caries in the primary dentition.

#### 3.3.2 AI machine learning (DMFT)

After comparison between the traditional logistic regression, ML logistic regression, XGBoost, Lasso, and Support Vector Machine methods, it was seen that the traditional logistic regression performed better in ROC AUC, accuracy, and Kappa statistic. Whereas XGBoost performed better in sensitivity measurement, and Lasso performed better in specificity measurement (Table 5).

**Table 5:** Model comparison between traditional models and ML (DMFT and deft).

Model	AUC-ROC	Accuracy	Sensitivity	Specificity	Kappa
FOR DMFT					
Traditional logistic regression	**0** **.** **76**	**0**.**70**	0.71	0.69	**0**.**40**
Machine learning: Logistic regression	0.74	0.68	0.66	0.69	0.36
Lasso regression	0.74	0.68	0.67	**0**.**70**	0.37
Support vector machine	0.75	0.69	0.69	0.68	0.38
XGBoost	0.75	0.69	**0**.**77**	0.61	0.38
FOR deft					
Traditional logistic regression	**0**.**87**	**0**.**81**	0.83	0.77	0.60
Machine learning: Logistic regression	0.86	0.78	0.71	0.83	0.55
Lasso regression	0.86	0.79	0.72	**0**.**84**	0.57
Support vector machine	0.86	0.79	0.70	**0**.**84**	0.56
XGBoost	0.86	**0**.**81**	**0**.**84**	0.79	**0**.**61**

Bold values are statistically significant.

#### 3.3.3 AI machine learning (deft)

After comparison between the traditional logistic regression, ML logistic regression, XGBoost, Lasso, and Support Vector Machine methods, it was seen that the traditional logistic regression performed better in ROC AUC. XGBoost and traditional logistic regression performed equally in accuracy, whereas XGBoost performed better in sensitivity measurement and Kappa statistic. Lasso and Support Vector Machine performed equally in specificity measurement (Table 5).

### 3.4 Comparison of variables’ importance

The assessment of variable importance was similar for both classical and machine learning algorithms. For primary dental caries, age ranked the highest followed by visible plaque and special needs. For permanent dentition, sugary meals consumption followed by plaque and insurance were considered the most valuable predictors.

## 4 Discussion

Dental caries continues to remain a huge concern for dentists, dental public health professionals, and patients as it is the most prevalent oral disease affecting children and adolescents resulting in deterioration of oral health ultimately leading to tooth loss (1). Although CRA has been highly recommended in clinical practice for management of dental caries, it is however severely underutilized by practitioners (7). Apart from CAMBRA, the other current systems are lacking validation. Therefore, development of future tools such as ML that can help utilize CRA items for better risk calculation and clinical outcomes prediction by implementing evidence-based caries management will tremendously help clinical practitioners. While many studies have been done in the past looking into dental caries predictors, these studies have been conducted using traditional statistical tools (4). There are no existing studies that have compared traditional statistical methods vs. ML in predicting dental caries.

Previous studies done on prediction of childhood dental caries showed that the presence of thick and heavy plaque is a predictor for caries development, progression, and activity (8). A study conducted by Lin et al. (9) showed that age was a useful predictor of childhood dental caries. Children with special needs were more likely to have dental caries as reported (10). Our study too found age, visible plaque, and special needs as strong predictors of dental caries comparable with previous studies conducted. Although sugary meals and fluoride exposure were not found to be significantly associated, they were part of the final model with best fit as seen in the literature (11). A previous study done on the prediction of dental caries for adolescents and adults showed poor oral hygiene and socioeconomic status as useful predictors (12). Our study showed that not having dental insurance was associated with dental caries.

Previous studies on childhood dental caries prediction utilizing ML solely found their proposed model yielded an AUC-ROC value of 0.74, sensitivity of 0.67, and accuracy of 0.64 (13). Another study (4) performed on children reported an AUC-ROC value of 0.78 for ML Logistic Regression, 0.785 for XGBoost, and 0.780 for Support Vector Machine. Our prediction data for children yielded an AUC-ROC value of 0.87 for traditional Logistic regression and an AUC-ROC value of 0.86 for ML (Logistic Regression, Lasso, XGBoost, and Support Vector Machine) outperforming (4) AUC-ROC value. Our study also showed a sensitivity of 0.84 for XGBoost and accuracy of 0.81 for XGBoost outperforming Karhade (13) values. Some of the limitations of the current study included the lack of generalizability to other populations. Data were obtained at Temple University Pediatrics, which serves a very high proportion of low-income African Americans in an urban setting.

Our prediction data on permanent dentition yielded AUC-ROC, accuracy, and Kappa values of 0.76, 0.70, and 0.40, respectively, for traditional Logistic Regression. Our study showed a sensitivity value of 0.77 for XGBoost and specificity value of 0.70 for Lasso. No prior studies comparing traditional vs. ML have been done on dental caries prediction of adolescents and adults.

## 5 Conclusions

Understanding the predictors of dental caries plays a huge role in reducing the burden of dental caries in this population. This study contributes to reducing the gap in literature about the role of various predictors on dental caries and the utilization of ML in the prediction. Both ML and the traditional statistical tool were able to generate predictors of dental caries. However, the ML model outperformed the traditional statistical model for primary caries prediction. The ML model had better accuracy, sensitivity, specificity, and Kappa values in comparison with the traditional statistical method. Thus, with confidence we can say that ML is an accurate, precise, and more meaningful statistical method that can be used for enhancing dental caries risk assessment. Simultaneously, these predictors can be used in day-to-day practice for aiding in clinical decision-making processes and for disease prevention in individual patients.

## Data Availability

The data analyzed in this study are subject to the following licenses/restrictions: The data that support the findings of this study are available on request from the corresponding author. The data are not publicly available due to privacy or ethical restrictions. Requests to access these datasets should be directed to priya.dey@temple.edu.

## Appendix A

**Table:** List of variables with explanations

Variables	Explanations	Categories
Fluoride exposure	Fluoride exposure (through drinking water, supplements, professional applications, toothpaste)	Yes = 1, No = 0
Sugary foods or drinks	Sugary foods or drinks between meals (incl. juice, soft drinks, energy drinks, medicinal syrups)	
Caries experience of family	Caries experience of mother, caregiver, and/or other siblings for patients aged 6–14	
Dental home	Dental home: established patient of record, receiving regular dental care in a dental office	Yes = 1, No = 0
Special healthcare needs	Special healthcare needs (developmental, physical, medical, or mental disabilities that prevent or limit performance of adequate oral healthcare by themselves or caregivers)	Yes = 1, No = 0
Medications that reduce salivary flow	Medications that reduce salivary flow	Yes = 1, No = 0
Visible plaque	Visible plaque as charted by the clinicians in the clinic at Kornberg School of Dentistry	Yes = 1, No = 0
Unusual tooth morphology	Unusual tooth morphology that compromises oral hygiene	Yes = 1, No = 0
Exposed root surfaces	Exposed root surfaces present as charted by the clinicians in the clinic at Kornberg School of Dentistry	Yes = 1, No = 0
Dental/orthodontic appliances	Dental/orthodontic appliances, fixed or removable	Yes = 1, No = 0
Severe dry mouth	Severe dry mouth (Xerostomia)	Yes = 1, No = 0
	Overall assessment of dental caries risk	
	Was caries prevention educational material provided to the patient?	Yes = 1, No = 0
	Patient has been offered fluoride application and declines to receive it in the clinic	Yes = 1, No = 0
